# The therapeutic potential of cyanidin-3-O-glucoside relating to female reproductive health

**DOI:** 10.3389/fphar.2025.1599688

**Published:** 2025-07-17

**Authors:** Katarina Majerik Behinska, Ema Balkova, Michal Mihal, Shubhadeep Roychoudhury, Adriana Kolesarova

**Affiliations:** ^1^ Institute of Applied Biology, Faculty of Biotechnology and Food Sciences, Slovak University of Agriculture in Nitra, Nitra, Slovakia; ^2^ AgroBioTech Research Centre, Slovak University of Agriculture in Nitra, Nitra, Slovakia; ^3^ Department of Life Science and Bioinformatics, Assam University, Silchar, India

**Keywords:** anthocyanin, reproductive disorders, ovary, hormone, apoptosis

## Abstract

Cyanidin-3-O-glucoside (C3G), a dietary flavonoid found in berries, exhibits strong antioxidant, anti-inflammatory, and anticancer properties. It plays a role in female reproductive health by protecting ovarian cells from oxidative stress while inhibiting tumour growth and inducing apoptosis in ovarian and cervical cancer cells. C3G can modulate estrogen receptors, growth factors, and apoptosis- and angiogenesis-related pathways. Its antioxidant and anti-inflammatory properties may be beneficial in hormone-related reproductive disorders and in oncological conditions of reproductive organs, such as ovarian cancer. Beyond its anticancer effects, C3G may be able to mitigate reproductive disorders such as polycystic ovary syndrome (PCOS), although its low bioavailability and need for improved delivery methods pose challenges. C3G influences gut microbiota and enhances systemic antioxidant activity, too. This evidence-based study summarizes the biological effects of C3G, emphasizing its impact on female reproductive health, proposing its mechanism(s) of action, and potential clinical application. Future pre-clinical and clinical investigations are needed to determine C3G’s effective dosages and assessment as a complementary or alternative therapy in gynecological oncology and reproductive health. Moreover, as many of these observations in the literature are based on large *in vitro* and enzyme-based studies that may be influenced by pan assay interference–a common challenge with some polyphenolic metabolites, such as C3G, the results must be interpreted with caution, and further *in vivo*, preclinical, and clinical investigations employing orthogonal and physiologically relevant approaches are warranted.

## 1 Introduction

Ovarian cancer is a significant challenge in gynecology, resulting in high mortality despite advances in treatment. In 2020, it was among the top eight cancers diagnosed in women, comprising 3.7% cases and 4.7% deaths. The 5-year survival rate ranged from 90% for localized cases to under 30% for advanced cancer cases (Webb and Jordan, 2024). This highlights the urgent need for innovative treatment strategies and therapeutic agents to improve patient outcomes.

Oxidative stress and inflammation contribute to reproductive diseases, including cancer of reproductive organs (Hu et al., 2020). Research on reproductive health is increasingly focusing on emerging extracellular and intracellular regulators that influence biological processes, including secretion, proliferation, differentiation, and apoptosis. These are vital to female reproductive health, as they influence ovarian function, folliculogenesis, and sexual development (Environmental Contaminants and Medicinal Plants, 2020).

Recently, naturally derived bioactive metabolites have been explored as alternative therapies for cancer treatment, including apigenin, quercetin, piperine, curcumin, and resveratrol (Auti et al., 2024). Bioactive flavonoids may activate tumour suppressors, inhibit angiogenesis, and reduce inflammation, bolstering their use in cancer prevention (Muniraj et al., 2019). Anthocyanins are particularly noted for their anti-cancer properties (Dharmawansa et al., 2020). Cyanidin-3-O-glucoside (C3G) exhibits various pharmacological effects, including antioxidant and anticancer properties (Liang et al., 2021). This anthocyanin, found abundantly in fruits and berries, inhibits cell proliferation and induces apoptosis in several types of cancers, including breast, colon, and prostate (Li et al., 2021a; Zeng et al., 2012; Sun et al., 2023). This evidence-based study highlights the unique therapeutic potential of C3G in the context of female reproductive health–an area where targeted natural interventions still remain underexplored. While anthocyanins have been widely studied for their general antioxidant and anticancer properties, their specific relevance to ovarian and uterine function has not been comprehensively summarized. This study provides a scientific basis for further investigation of C3G as a promising adjunct agent in the prevention and/or management of reproductive disorders, including cancers of reproductive organs.

## 2 Methodology

A structured literature search was conducted to identify relevant studies on the biological and therapeutic effects of C3G on female reproductive health. The keywords used for the search in PubMed and SCOPUS databases included “cyanidin-3-O-glucoside,” “anthocyanin,” “female reproduction,” and “anticancer activity.” The search revealed a total of 640 articles published during the last 10 years, i.e., 2015 to 2024, out of which a total of 33 were selected based on their focus on the properties and potential roles of C3G in women’s health. Articles published in languages other than English were also excluded.

## 3 Chemical properties and bioavailability

Fruits are rich in dietary anthocyanins with concentrations up to 1 g per 100 g. C3G (C_21_H_21_O_11_, 449.39 g/mol) contains a cyanidin aglycone linked to glucose at the 3-position of the C-ring (Liang et al., 2021; Olivas-Aguirre et al., 2016). Its colour originates from a stable flavylium cation structure under acidic conditions (pH < 4), but can degrade in neutral or alkaline environments. Antioxidant properties stem from the hydroxyl-rich B-ring, which aids in free radical scavenging and metal ion chelation (Galvano et al., 2004). C3G absorbs light at 520–540 nm, enhancing its characteristics. It is sensitive to light, temperature, oxygen, and pH, interacting with proteins and DNA via hydrogen bonding, which can influence cellular pathways and gene expression. After ingestion, it metabolizes into phenolic acids, maintaining systemic antioxidant effects crucial for its therapeutic potential (Liang et al., 2021; Olivas-Aguirre et al., 2016; Caprioli et al., 2016).

## 4 Metabolism

The average dietary intake of anthocyanins, including C3G, ranges from 1.9 to 74.6 mg per day, primarily sourced from berries, grapes, cherries, and certain vegetables such as red cabbage (Kimble et al., 2019). Upon ingestion, C3G is absorbed through the gastrointestinal tract with an efficiency of approximately 4% in animals and up to 12% in humans (Olivas-Aguirre et al., 2016; Galvano et al., 2004). In the bloodstream, it undergoes phase I and II metabolism, where enzymes in the small intestine cleave the glucose moiety, releasing the aglycone cyanidin. This intermediate is subsequently converted into phenolic acids such as protocatechuic acid (PCA), which serves as a major bioactive metabolite in humans (Galvano et al., 2004; Tan et al., 2019).

Additionally, microbial metabolism in the distal small and large intestine contributes to the breakdown of the heterocyclic flavylium (C-ring) via dehydroxylation and decarboxylation, generating metabolites like ferulic acid, vanillic acid, and caffeic acid (Tan et al., 2019). These metabolites are absorbed into systemic circulation and further processed in the liver and kidneys, including via enterohepatic recycling. Although present in low concentrations in plasma and urine, these metabolites are biologically active and rapidly processed (Galvano et al., 2004; Tan et al., 2019). Their activity is associated with lowering oxidative stress, modulating inflammation, and supporting intestinal barrier function. The involvement of gut microbiota in these metabolic transformations highlights the complex interaction between dietary anthocyanins and host physiology (Cheng et al., 2022; Wang et al., 2022). Despite its promising biological effects, the clinical potential of C3G is limited by its low bioavailability. Recent studies have explored formulation strategies such as nanoencapsulation, liposomal delivery, or co-administration with absorption enhancers to improve its pharmacokinetic profile and therapeutic efficacy. For instance, nano-formulations have been shown to enhance the stability and bioavailability of anthocyanins (Matsumoto et al., 2007; Rashwan et al., 2023). Additionally, co-administration with metabolites like phytic acid has been reported to improve the absorption of anthocyanins in both animal models and humans.

## 5 Effects on female reproduction

### 5.1 Effects on ovarian cells

C3G exhibits antiproliferative and pro-apoptotic effects on ovarian cancer cells, with IC50 values ranging from 10 to 15 mg/L (Zeng et al., 2012). It interacts with estrogen receptor beta (ERβ), triggering apoptosis via caspase activation and downregulating oncogenic factors, such as mucin 4 (MUC4), which is linked to metastasis (Liu et al., 2019). Anthocyanin-rich mixtures (which include C3G as a metabolite) also reduce the proliferation of ovarian cancer cells in specific cell lines by lowering integrin expression, which is essential for cell adhesion and migration, and inhibiting vascular endothelial growth factor receptor-3 (VEGFR-3) phosphorylation, a crucial step in tumour vascularization (Aqil et al., 2017; Teller et al., 2009). *In vivo* studies suggest that C3G may offer a potential adjuvant therapy, reducing tumour growth in ovarian cancer models via oral administration (Wang et al., 2022) (Table 1). A recent study on non-cancerous granulosa cell culture has reported C3G’s ability to mitigate apoptosis induced by zearalenone, an estrogenic mycotoxin, through activation of phosphatidylinositol 3-kinase/protein kinase B (PI3K/AKT) pathway (Li et al., 2023). In a rat model of ovarian damage caused by cadmium exposure, C3G treatment lowered oxidative stress and restored antioxidant enzyme activity, including glutathione peroxidase (GPX) and superoxide dismutase (SOD), thereby preserving ovarian function (Yang et al., 2022). In a model of ovarian torsion and detorsion, anthocyanin administration also lowered oxidative stress and tissue damage, thus protecting against follicular degeneration and supporting ovarian function (Teymoori et al., 2023). These findings highlight C3G’s dual role in both inhibiting ovarian cancer and preserving ovarian function.

**TABLE 1:** Action of anthocyanins, including cyanidin-3-O-glucoside (C3G), on female reproductive processes.

*In vitro*			
Experimental model	Active principle	Mechanism(s) of action	References
ERα36-positive breast cancer cells	C3G	Binds to ERα36 and inhibits cancer proliferation	Wang et al. (2017)
Ovarian cancer cell line (HO-8910PM)	C3G	Induces apoptosis and reduces MUC4 expression, thereby inhibiting the growth of ovarian tumours	Zeng et al. (2012)
Human vulva carcinoma cell line (A431)	C3G, delphinidin-3-glucoside, peonidin-3-glucoside, malvidin	↓ RTKs, ↓ VEGFR-3 phosphorylation, ↓ ErbB3 activity	Teller et al. (2009)
Human cervical tumour cells (HeLa)	Chokeberry extract	↑ Induced apoptosis, ↑ ROS scavenging and oxidative stress reduction	Rugină et al. (2012)
Ovarian cancer cell line (A2780, A2780/CP70, OVCA432, OVCA433)	Anthocyanidins: delphinidin, cyanidin, malvidin, peonidin, petunidin	↓ Growth of ovarian cancer cells, ↑ antiproliferative activity, ↓ p-glycoproteins in OVCA432 cells	(Aqil et al., 2017)
Granulosa cell culture (porcine)	C3G	Protects against zearalenone-induced apoptosis by restoring PI3K/AKT-mediated survival signaling	Li et al. (2023)

*In vivo*			
Xenograft mouse model of ovarian cancer	C3G	Reduces tumour growth and oncogenic pathways, supporting adjuvant therapy	Wang et al. (2022)
Mouse model of ovarian toxicity	C3G and zearalenone	Prevents ovarian damage caused by environmental toxins by modulating the p53/GADD45a pathway	Zhang et al. (2023)
Mouse uterus	C3G and cadmium	↓ Cadmium-induced uterine epithelium proliferation	Yang et al. (2022)
Mouse model of menopause-induced ovarian aging	Anthocyanins from purple sweet potato	Delays ovarian aging by reducing oxidative stress and preserving follicle reserve	Li et al. (2018)
Ovarian damage and oxidative stress markers in adult female rat after torsion/detorsion	Anthocyanins (generic term)	Reduction of oxidative stress markers in ovarian damage	Teymoori et al. (2023)
Rat model of uterine inflammation	Anthocyanin-rich berry extract	Reduces uterine inflammation by modulating NF-kB signaling and lowering pro-inflammatory cytokines	Li et al. (2021b)

### 5.2 Effects on uterine function

C3G shows promise in uterine health, particularly in cervical cancer and endometrial receptivity. In HeLa cells, C3G together with cisplatin decreased the expression of nuclear factor erythroid 2-related factor 2 (Nrf2), thereby reducing the activity of antioxidant enzymes such as heme oxygenase-1 (HO-1) and NAD(P)H quinone oxidoreductase 1 (NQO1). An increase in oxidative stress led to apoptosis, highlighting C3G’s potential as a chemotherapeutic agent (Li et al., 2021b). C3G interacts with estrogen receptors in cervical tumour cells, affecting nuclear factor kappa-B (NF-κB) and Nrf2 pathways, which are crucial in managing oxidative stress and cell viability. Anthocyanin-rich chokeberry extracts (which contain C3G as a metabolite) also possess a similar protective effect by scavenging reactive oxygen species (ROS) in reproductive tissues (Rugină et al., 2012). C3G supports uterine function by fostering an anti-inflammatory environment vital for endometrial receptivity. By modulating inflammatory mediators, C3G may create favourable conditions for implantation and reduce the failure risk associated with oxidative stress and chronic inflammation (Yang et al., 2022).

## 6 Discussion

**FIGURE 1:**
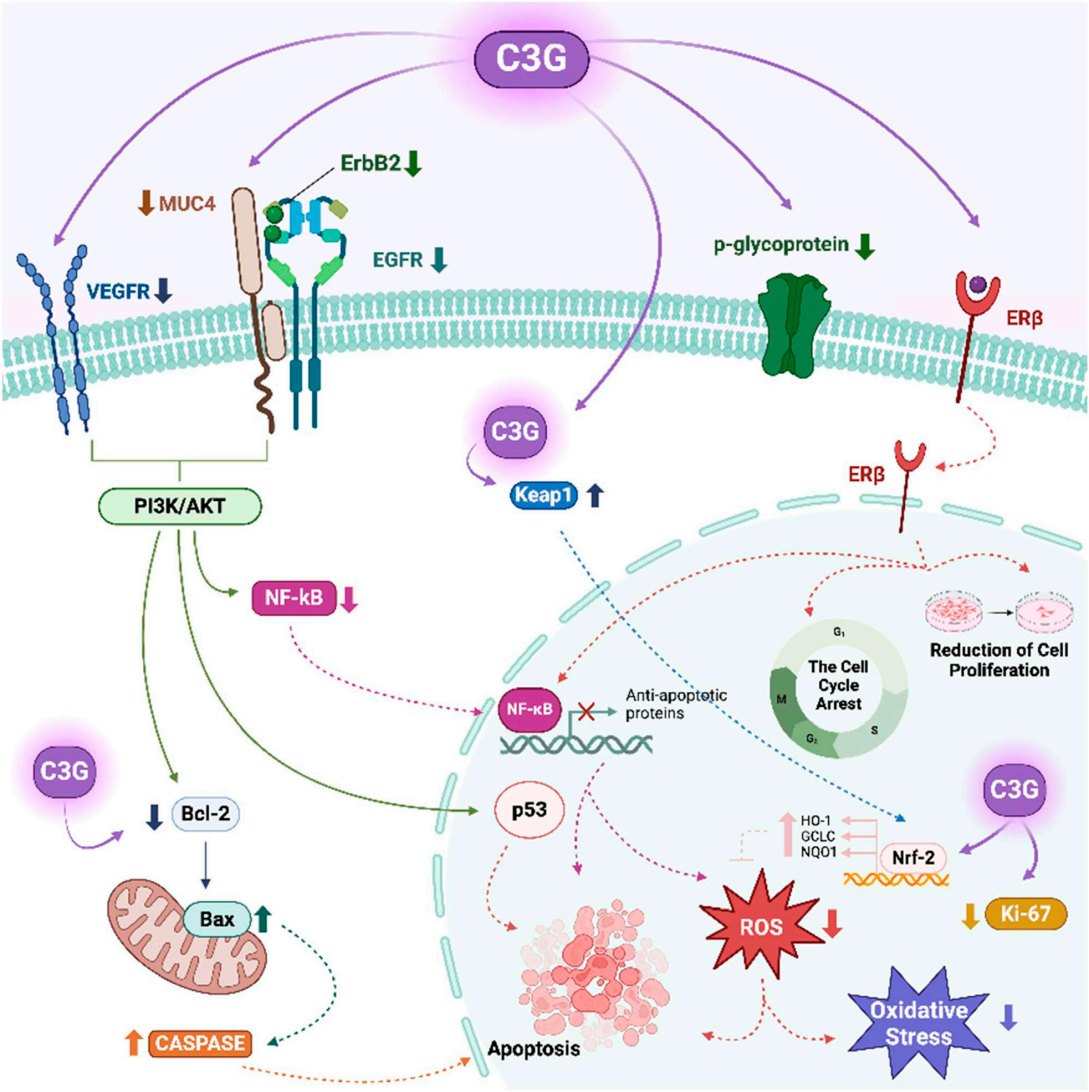
Apoptotic effect of cyanidin-3-O-glycoside (C3G) via multiple cellular signaling pathways on female reproductive processes. Created in BioRender. Kolesárová, **(A)** (2025) https://BioRender.com/r40y023. Bax: Bcl-2-associated X protein, Bcl-2: B-cell lymphoma 2, Caspase: Cysteine aspartate protease, C3G: Cyanidin-3-O-glucoside, EGFR–Epidermal growth factor receptor, Erβ: Estrogen receptor beta, ErbB2: Receptor tyrosine kinase 2, GCLC: Glutamate-cysteine ligase catalytic subunit, HO-1: Heme oxygenase-1, Keap1: Kelch-like ECH-associated protein 1, Ki-67: Marker of proliferation Ki-67, NQO1: NAD(P)H quinone dehydrogenase 1, MUC4: Mucin 4, NF-κB–Nuclear factor kappa B, Nrf-2: Nuclear factor erythroid 2-related factor 2, VEGFR: Vascular endothelial growth factor receptor, p53: Tumour protein p53, p-glycoprotein: Permeability glycoprotein, PI3K/AKT–Phosphoinositide 3-kinase/Protein kinase B (AKT) pathway, ROS: Reactive oxygen species.

C3G exerts biological effects via its antioxidant, anti-inflammatory, and estrogen receptor-modulating properties. *In vitro* and *in vivo* studies provide significant information about C3G’s effects on the female reproductive system (Table 1). C3G demonstrates selective affinity for ERβ, which is associated with tumour suppression, apoptosis induction, and anti-inflammatory effects in ovarian cancer cells (Nanashima et al., 2018). Selective modulation of ERβ is pharmacologically relevant, as ERβ activation has been associated with anti-proliferative and anti-inflammatory effects, contrasting the mitogenic role often linked to ERα. From a clinical perspective, targeting ERβ may offer therapeutic benefits in hormone-sensitive cancers and inflammatory reproductive disorders while minimizing risks associated with generalized estrogenic stimulation. This receptor selectivity makes C3G a promising candidate for safer endocrine modulation in female health. It also downregulates the PI3K/AKT pathway, reducing survival signaling and promoting pro-apoptotic mechanisms (Li et al., 2021b). Additionally, it induces G2/M phase arrest by suppressing cyclin B1, which may affect anticancer effects on ovarian cells (Wang et al., 2017). In HO-8910 p.m. ovarian cancer cells, C3G inhibits proliferation and induces apoptosis by downregulating mucin1. C3G also stimulates Nrf2 pathway, enhancing antioxidant defense and vital enzymes like SOD, catalase (CAT), and GPx (Zeng et al., 2012; Rahman et al., 2021). These enzymes repair DNA damage and influence DNA repair through Ataxia-Telangiectasia Mutation (ATM) and p53 proteins, thereby reducing cell death caused by oxidative stress. This highlights C3G’s potential as a novel cancer therapy. Additionally, C3G scavenges ROS, alleviates antioxidant enzyme activity, modulates the ERK/Nrf2 pathway, and protects against oxidative damage (Rahman et al., 2021). It is also crucial for angiogenesis, vascularization, and endometrial receptivity. C3G activates the PI3K/Akt pathway, promoting cell survival and vascular growth in the endometrium (Li et al., 2023). It can inhibit receptor tyrosine kinases (RTKs), such as VEGFR-2, VEGFR-3, epidermal growth factor receptor (EGFR), and human epidermal growth factor receptor 3 (ErbB3) that are essential for angiogenesis and tumour proliferation. C3G also impacts female reproductive health through modulation of estrogen receptors, antioxidative property, regulation of angiogenesis, and induction of apoptosis (Sivasinprasasn et al., 2016). Its modulation of the Bax/Bcl-2 ratio suggests a mitochondrial apoptotic mechanism, as noted in cancer but less understood in normal reproductive tissues (Tan et al., 2019). The proposed apoptotic mechanisms of C3G in ovarian cells are summarized in Figure 1. It illustrates the modulation of key signaling pathways involved in apoptosis, including upregulation of pro-apoptotic proteins such as Bax, downregulation of anti-apoptotic Bcl-2, and subsequent activation of caspase cascades. These effects are supported by previous findings demonstrating C3G’s ability to influence intrinsic apoptotic pathways and to modulate the expression of apoptosis-related genes and proteins in cancer cells.

## 7 Potential clinical application

Given its favourable biological profile, C3G poses as a suitable candidate for future clinical application in female reproductive medicine. It's demonstrated ability to reduce tumour proliferation in ovarian and cervical cancers (Sun et al., 2023; Wang et al., 2022), to attenuate oxidative and inflammatory damage in the uterus and ovaries (Li et al., 2023; Yang et al., 2022; Li et al., 2018), and to modulate hormonal and signaling pathways such as PI3K/AKT and ERβ-mediated signaling (Liu et al., 2019; Li et al., 2023) supports its potential in managing both oncological and endocrine-related reproductive disorders. Beyond reproductive health, C3G exerts systemic antioxidant and anti-inflammatory effects, which contribute to its overall therapeutic value across various organ systems (Rahman et al., 2021). These pleiotropic actions make it a promising complementary agent for improving health outcomes in conditions linked to oxidative stress, inflammation, and hormonal imbalance. Although its clinical application is currently limited by low oral bioavailability, emerging formulation technologies such as nanoencapsulation, liposomal delivery, and co-administration with absorption enhancers (e.g., phytic acid) offer promising solutions to enhance its stability and systemic exposure (Matsumoto et al., 2007; Rashwan et al., 2023; Aqil et al., 2017). These strategies can serve as supportive tools to ensure sufficient therapeutic levels without compromising the metabolite’s safety or natural origin. Available toxicological data indicate that C3G is generally considered safe, with no significant adverse effects reported at physiologically relevant doses in animal models or human observational studies (Olivas-Aguirre et al., 2016; Galvano et al., 2004). Long-term consumption of anthocyanin-rich foods has not been associated with toxicity. However, more comprehensive safety assessments are required to confirm its suitability for chronic therapeutic use. Future studies should focus on the validation of C3G’s effects in human clinical trials, particularly in women suffering from hormone-related reproductive conditions or undergoing treatment for cancers of reproductive organs. This may include establishing optimal dosing regimens, long-term safety profiles, and exploring its integration into nutraceutical or adjuvant oncological therapies. Although numerous *in vitro* studies, as discussed above, highlight the pharmacological potential of C3G, it is crucial to acknowledge that many polyphenolic metabolites, including C3G, are prone to pan-assay interference (PAINS). Such interference may lead to false positives by nonspecific binding, aggregation, or other assay artifacts (Bolz et al., 2021). As recommended in the literature, *in vitro* results should be interpreted with caution and supplemented by orthogonal approaches that better approximate physiological conditions (Magalhães et al., 2021). Thus, while the data reviewed herein support the therapeutic promise of C3G, further studies employing rigorous pharmacokinetic and *in vivo* analyses are necessary to confirm the clinical relevance.

## 8 Conclusion

C3G shows promising potential for enhancing female reproductive health mainly owing to antioxidant and anti-inflammatory properties, ability to reduce oxidative stress, and pro-apoptotic effects on cancer cells. However, many of these observations are based on large *in vitro* and enzyme-based studies that may be influenced by PAINS, a common challenge with some polyphenolic metabolites, such as C3G. Therefore, even though the evidence is compelling, the results must be interpreted with caution, and further *in vivo*, preclinical and clinical investigations employing orthogonal and physiologically relevant approaches are required. Such studies will be essential to confirm the therapeutic efficacy, determine effective dosages, and assess the clinical relevance of C3G as a complementary or alternative therapy in gynecological oncology and reproductive health.
